# Comparing Contact Tracing Through Bluetooth and GPS Surveillance Data: Simulation-Driven Approach

**DOI:** 10.2196/38170

**Published:** 2024-04-17

**Authors:** Weicheng Qian, Aranock Cooke, Kevin Gordon Stanley, Nathaniel David Osgood

**Affiliations:** 1 Department of Computer Science University of Saskatchewan Saskatoon, SK Canada; 2 Department of Community Health & Epidemiology University of Saskatchewan Saskatoon, SK Canada; 3 Bioengineering Division University of Saskatchewan Saskatoon, SK Canada

**Keywords:** smartphone-based sensing, proximity contact data, transmission models, agent-based simulation, health informatics, mobile phone

## Abstract

**Background:**

Accurate and responsive epidemiological simulations of epidemic outbreaks inform decision-making to mitigate the impact of pandemics. These simulations must be grounded in quantities derived from measurements, among which the parameters associated with contacts between individuals are notoriously difficult to estimate. Digital contact tracing data, such as those provided by Bluetooth beaconing or GPS colocating, can provide more precise measures of contact than traditional methods based on direct observation or self-reporting. Both measurement modalities have shortcomings and are prone to false positives or negatives, as unmeasured environmental influences bias the data.

**Objective:**

We aim to compare GPS colocated versus Bluetooth beacon–derived proximity contact data for their impacts on transmission models’ results under community and types of diseases.

**Methods:**

We examined the contact patterns derived from 3 data sets collected in 2016, with participants comprising students and staff from the University of Saskatchewan in Canada. Each of these 3 data sets used both Bluetooth beaconing and GPS localization on smartphones running the Ethica Data (Avicenna Research) app to collect sensor data about every 5 minutes over a month. We compared the structure of contact networks inferred from proximity contact data collected with the modalities of GPS colocating and Bluetooth beaconing. We assessed the impact of sensing modalities on the simulation results of transmission models informed by proximate contacts derived from sensing data. Specifically, we compared the incidence number, attack rate, and individual infection risks across simulation results of agent-based susceptible-exposed-infectious-removed transmission models of 4 different contagious diseases. We have demonstrated their differences with violin plots, 2-tailed *t* tests, and Kullback-Leibler divergence.

**Results:**

Both network structure analyses show visually salient differences in proximity contact data collected between GPS colocating and Bluetooth beaconing, regardless of the underlying population. Significant differences were found for the estimated attack rate based on distance threshold, measurement modality, and simulated disease. This finding demonstrates that the sensor modality used to trace contact can have a significant impact on the expected propagation of a disease through a population. The violin plots of attack rate and Kullback-Leibler divergence of individual infection risks demonstrated discernible differences for different sensing modalities, regardless of the underlying population and diseases. The results of the *t* tests on attack rate between different sensing modalities were mostly significant (*P*<.001).

**Conclusions:**

We show that the contact networks generated from these 2 measurement modalities are different and generate significantly different attack rates across multiple data sets and pathogens. While both modalities offer higher-resolution portraits of contact behavior than is possible with most traditional contact measures, the differential impact of measurement modality on the simulation outcome cannot be ignored and must be addressed in studies only using a single measure of contact in the future.

## Introduction

### Sensing Modality of Colocation for Disease Transmission Models

Infectious diseases have imposed a heavy burden on the global population throughout human history [1,2]. The COVID-19 pandemic has brought the threat of contagious diseases into sharp focus. With 6.95 million deaths; 769 million confirmed cases globally as of August 8, 2023 [3]; and an estimated >US $16 trillion in lost economic activity [4] for the United States alone, the COVID-19 pandemic has been one of the defining global crises of the 21st century [5].

Epidemiological models date back over a century [6-8] but have become more useful through leveraging sophisticated algorithms [9,10] and increasing computing power [11,12]. Epidemiological models to predict, plan, and respond to pandemics and outbreaks can inform decision-making to mitigate the impact of infectious diseases [6-8]. Epidemiological models require well-grounded physical and behavioral factors to provide reasonable estimates of disease spread [13]. Linking a population’s spatial behavior to infectious events that aids or inhibits the spread of disease is particularly difficult.

For airborne contagious diseases such as measles [14] or COVID-19 [5], a key enabler for disease spread is colocation (being in the same location at the same time). The effective spatial volume for COVID-19 is determined by aerosol dynamics and is often approximated at 2 m (6.56 ft) [15]. Measuring colocation can be conducted by self-reporting, as is commonly used in classic contact tracing and direct observation and counts [16], or by electronic means [17-19].

A total of 2 primary modalities for determining colocation using electronic devices exist: measurements based on estimating the distance from one person to another directly (beaconing) and measurements based on estimating the location of each person of interest within a coordinate system and calculating distances (localizing). Devices can be bespoke, such as the sociometric badge [20-22], or can leverage existing technologies such as Bluetooth(BT)–enabled phones, beacons, or dongles [23-26]. Localization techniques use systems such as GPS to place every user at a specific location at a specific time [27,28] and can be piggybacked on existing smartphones or mined from some social media platforms [29-31].

To estimate the probabilities of disease transmission, the total number of interactions, dwell times, and spatial proximity must be measured to properly baseline the parameter estimates. Techniques from companies such as Ethica Data (Avicenna Research) [32] and other companies made possible by a Google-Apple partnership [33] can be used to obtain these data for target populations under transparent and ethical data acquisition practices. However, the underlying physical processes and mathematical treatment of beaconing and localization data are substantially different and have different failure modes. Previous research had not elucidated the disparities in the estimated contact patterns for the same population between techniques. It is simple to hypothesize that colocating and beaconing will yield different contact patterns, but it is less apparent how the differences will interact with disease dynamics and impact the overall simulation outcomes.

In this study, we examine the contact patterns derived from 3 previously collected data sets using both BT beaconing and GPS localization on smartphones running the Ethica Data app. We demonstrated that while the underlying contact patterns generated from colocating and beaconing are broadly similar, they contain salient differences. For each of the 4 pathogens marked by different dynamics, we compared the results of an agent-based simulation of a communicable disease outbreak for the pathogen parameterized with beaconing- and localization-derived contact patterns. The results demonstrated that the method used to estimate contact patterns can result in significant differences between estimates of the key outbreak parameters. We showed that GPS-based contact patterns estimate significantly fewer and less-severe outbreaks than BT-derived contact patterns for the same participant and device.

### Literature Review

Transmission models for communicable diseases are based on the characterization of natural history of a condition and contact networks [13]. In addition to traditional population-based nonspatial approaches, agent-based transmission models can use individual-level contact records and behaviors to identify emergent patterns using a bottom-up approach [9].

Real-world proximity tracking has applications in contact tracing, location-based risk assessment, mobility tracking, and outbreak detection [34]. Deriving real-world proximity contact mainly falls into 2 categories: calculating the delta of measured absolute positions—with, for example, GPS-assisted and Wi-Fi network–assisted locationing [35,36]—and directly measuring the relative distance with, for example, BT [19,37] or radio frequency identification [38].

Exemplars of each of these 2 approaches—GPS and BT—have been studied for digital contact tracing and transmission simulation [15,34]. Recent comparisons between GPS- and BT-inferred proximity contact collection approaches focus on privacy preservation, adoption, and compliance rates [15]. In contrast, the accuracy of simulations with GPS- and BT-derived proximity contacts is yet to be quantified across different underlying populations and pathogens [37].

Advances in digital contact tracing have also contributed to disease parameter estimation. For example, at the beginning of the COVID-19 pandemic, researchers focused on estimating the basic reproduction number *R_0_* from limited and highly regionally dependent infection data. As the pandemic spread, data collection and reporting standards enabled the daily reporting of incident cases, active cases, and mortality for various geographic scales over time, allowing the estimation of the effective reproduction number *R_e_* [39-42].

### Background

#### BT Proximity

BT is a short-range communications protocol incorporated into most smartphones and is commonly used to pair with devices such as wireless headsets. By default, BT is configured to be in a quiescent state, not advertising its presence and only communicating with devices that have been paired. Before 2020, it was possible to lock an Android phone into a more active discovery mode, where a device would beacon approximately every 8 seconds, advertising its presence to other devices. While this functionality was intended to provide ease of initial device pairing, it could be repurposed to detect the proximity of 2 devices by registering when 1 device receives a discovery ping from another.

Studies [43,44] have investigated the use of BT to estimate the spatial proximity between devices representing people. The simplest methodology will be to create a proximity event between 2 devices if 1 device detects a discovery ping from the other or vice versa. The distance between the devices is a relevant parameter for determining a valid proximity event or contact in many applications. Researchers have typically used the Received Signal Strength Indicator (RSSI) as a proxy for distance [44-46], assuming an exponential falloff of signal strength with distance [47,48]. This approximation is confounded by reflections or transmissions off or through objects, meaning that RSSI cannot be strictly interpreted as distance, except in all but the most controlled conditions. RSSI values can plausibly be used to filter out contacts that are either far away or on the other side of a barrier, such as a wall.

The RSSI measures signal strength in decibel-milliwatts (dBm), where RSSI=0 is defined by a “Golden Receiver Power Range,” whose lower threshold level corresponds to a received power between −56 dBm and 6 dB above the actual sensitivity of the receiver, and whose upper threshold level is 20 dB above the lower threshold level to an accuracy of 6 dB. Beyond the lower and upper threshold, any positive RSSI value indicates how many dB the RSSI is above the upper limit, and any negative value indicates how many dB the RSSI is below the lower limit. Usually, a stronger signal strength (higher RSSI) indicates closer distances between 2 BT devices; however, orientation, barriers, and interference can attenuate the signal strength beyond what the distance would suggest [49]. Young [50] and the Android Beacon Library [51] contributed an RSSI to the distance function based on the Nexus 4 and Apple’s iBeacon performance, which is often used as a first approximation for similar location awareness services on modern smartphones:



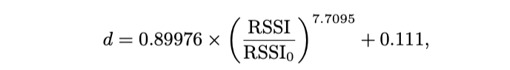



Where RSSI_0_ is the RSSI value at a 1-m (3.3 ft) distance.

#### GPS and Location Proximity

GPS receivers are standard on smartphones, enabling location-based services and route finding. Consumer-grade GPS receivers typically have a nominal accuracy of 10 m (32.8 ft) but can be subject to substantially larger errors due to environmental factors. Neither iOS nor Android uses pure GPS localization in their location estimation services. Both additionally use initial estimates from cell tower locations (assisted GPS) as well as fingerprinting-based localization using databases of detected Wi-Fi routers. As GPS receivers often take several seconds to obtain a position lock, even with assisted GPS, smartphone localization services tend to default to Wi-Fi–based localization initially and then switch to GPS as better location estimates become available. For simplicity of presentation, the term GPS refers to location estimation in this paper, regardless of whether it was obtained through GPS, assisted GPS, Wi-Fi fingerprinting, or some combination thereof.

Given location records, a dichotomous notion of proximity can be defined, in which 2 agents are considered proximate if they are in the same place at the same time. The precision and accuracy of the measurements and the context of the definition of proximity determine how close, in time and space, agents must be to be considered proximate or in contact. When using commodity smartphone localization hardware and services, accuracy below 5 m (16.4 ft) is rare [28], so spatial proximity has a strong lower resolution limit. Temporal resolution is substantially better—on the order of seconds—and is more likely to be limited by the measurement regime or application requirements. Elevation estimates are even less reliable than spatial estimates; therefore, commodity GPS receivers are often projected onto a 2D plane, introducing the potential for erroneous connections between people at the same location but on different floors of a building, for example.

While both GPS and BT can provide higher fidelity estimates of proximity and contact than traditional surveys or diaries, both are prone to false positives and negatives. Given 2 devices separated by a mutually proximate wall, ceiling, or floor, BT can still report contacts because the attenuation of RSSI will be such that they appear in contact but farther away. GPS is prone to false positives for detecting the proximity of communicable pathogens because the distance over which transmission can occur is smaller than the accuracy threshold for commodity devices. GPS proximity can only be interpreted as close enough that contact was possible, given the error in measurement, and not that contact actually occurred. BT can produce false negatives if the beaconing and listening cycles of the devices are misaligned, such that 1 device is beaconing while the other is asleep. GPS can lose signal or accuracy when indoors, causing false negative contacts by either having no location reported for an agent or exhibiting position inaccuracies that render inaccurate colocation calculations. While the underlying true contact dynamics for the same devices are identical, the differing failure modes of GPS and BT mean that data drawn from those data collection modalities may generate different contact networks, thereby suggesting different contact dynamics and ultimately different outbreak dynamics.

#### Agent-Based Susceptible-Exposed-Infectious-Removed Models

The susceptible-exposed-infectious-removed (SEIR) disease state model is a classic model used to characterize pathogen transmission and the natural history of infection across a range of communicable diseases. Disease state transitions are unidirectional in the order of susceptible, exposed, infectious, and removed. The initial state of the model specifies the amount of population in each disease state, and the rate of transition between disease states is subject to both disease-characteristic parameters (such as latent period and infectious period) and the contact network (such as preferential mixing and average contact rate). It is common for a specific disease, given surveillance data, to obtain more detailed models. For example, there are models of COVID-19 splitting the SEIR states into more states and rerouting transitions in states [52,53]. As our goal was to probe the impact of contact measurement modality, in accordance with the Occam's razor [54], which recommends a parsimonious model with the fewest assumptions that are necessary, we chose the simplest SEIR model.

Agent-based models (ABMs) incorporate individual interactions and track the state and state transitions through which each individual progresses. Unlike a stock and flow model, which uses differential equations to model the flow of individuals from one state to another in aggregate, an ABM knows the state of every agent individually at any time step of the simulation, and aggregate statistics, for example, on infections, are queried and computed during postprocessing. An agent-based SEIR model captures both individual disease state transitions based on disease-specific parameters such as the latent period, the infectious period, *R_0_*, as well as some abstraction of the contact behavior of the population. As the simulation of an infectious disease can capture emerging patterns in a bottom-up manner [9] and more faithfully reflect dynamics due to the proximity contact network than compartmental models, ABMs provide higher fidelity at the cost of computation when compared to stock and flow models. As an ABM can directly use a contact pattern as part of the simulation, it is the logical choice for examining the sensitivity of simulations to the contact detection methodology.

## Methods

### Data Set Description

For this study, we used 3 previously collected data sets, all of which were collected from the city of Saskatoon, a city in the midwestern Canadian province of Saskatchewan. In all these data sets, additional sensor modalities (eg, accelerometer, gyroscope, and Wi-Fi traces) were also collected, but only the BT traces, GPS traces, and battery data were used in this study. Battery data were used to identify gaps in data collection. If the phone is on and Ethica is running, then battery data were recorded, providing a more reliable way to assess the continuity of data collection than is possible with GPS, where signals can be obscured by the built environment but where the phone is still actively recording. The Saskatchewan Human Ethology Datasets (SHEDs) are a collection of pilot projects and technical trials taking place during the iEpi project—the academic precursor for the Ethica Data system—and associated postprocessing and methodological outcomes [55,56]. The SHEDs were exclusively collected from populations at the University of Saskatchewan in Saskatoon. The SHED7 data set was collected between July 11 and August 8, 2016, and included 61 students. The SHED8 data set was collected between September 25 and October 25, 2016, and included 74 students. The SHED9 data set was collected between October 28 and December 9, 2016, and included 88 students. These participants were part of a social science student study pool that included both undergraduate and graduate students and was weighted toward undergraduates.

### Ethical Considerations

Data collection and analysis were conducted under written approval (BEH-14-203) from the University of Saskatchewan Human Behavioral Ethics Review Board. All data were collected with the informed consent of the participants and under the oversight of the University of Saskatchewan Human Behavioral Ethics Review Board.

No experimental manipulations were conducted during data collection. The studies did not undertake stratified sampling according to ethnicity, grade, or gender. The study did not proscribe participation by those connected with the department or research laboratories involved, and the study team informed colleagues in laboratories and the Department of Computer Science first. The awareness of potential study involvement can be assumed to have spread across social networks. For each study, participants joined using their own phones to install the Ethica app and consented to have sensor data collected over the study period. Although for all these 3 studies, both Android and iPhone users were welcome, because BT beaconing did not work reliably on an iPhone due to security settings, iPhone users were removed from the analysis, and all participants reported here are Android users. Each participant receives approximately a CAD $50 (US $38.6) honorarium at the end of a study, and the exact amount varies by study length.

All 3 SHED studies stored sensor data anonymously, and the sensor data associated with a participant were identified by a device-identity number. Despite measures such as encryptions being used to protect sensor data, high-velocity GPS data can allow a skilled practitioner to determine salient information about participants, such as place of work, residence, and daily habits. We circumvented this issue by committing to our own research ethics board and the participants that researchers who access the data must commit to writing requests subject to the review and approval of our ethics boards.

### Sensor Data Processing

#### Overview

To evaluate the performance of each sensor in real-world scenarios, we needed to account for the impact of participant compliance. We defined the active period of a study with the start day as the first day when we have ≥80% of the participants’ battery reading and the end day as the first day with all following days having <80% of the participants’ battery reading. We retained participants who had at least 50% of the daily battery data. The descriptive statistics are presented in Table 1.

**Table 1:** Sensor data table for the 3 data sets (Saskatchewan Human Ethology Dataset [SHED]7, SHED8, and SHED9). These data sets are collected in 2016 with participants comprises students and staff of University of Saskatchewan. Each of these data sets is collected with the Ethica Data app with Bluetooth and GPS sensors with duty cycle about every 5 minutes over a month.

	SHED7	SHED8	SHED9
Participants, N	61	74	88
Retained participants, n	61	74	78
Total days in studies, n	35	31	41
Active days in studies, n	28	30	38
Bluetooth-inferred contacts (distance threshold: 8 m), n	37,804	34,400	20,597
GPS-inferred contacts (distance threshold: 10 m; accuracy: 10 m), n	4338	6784	5064

Ethica’s multisensor sensing requests that the Android operating system perform sensor scanning and reading periodically. We call our requested period length, that is, each of the repeated 5-minute time windows, a duty cycle. For the location and BT contact data used in this study, Ethica records for 1 minute, starting every 5 minutes.

The BT discovery record from the Android application programming interface includes the discovered BT device’s MAC address and RSSI. After linking such discovery records to participant IDs in the smartphone BT MAC address table collected after consent and before the experiment started, we created a table of BT discovery records for eligible participants. Those RSSI values were filtered to include records associated with an RSSI stronger than the RSSI values associated with the desired distance thresholds and then aggregated maximum RSSI values for unique tuples of discovered participant and duty cycle (data collection epoch), resulting in the final BT contact record table. Although BT discovery records are directional, our use of unique tuples will consider a pair of participants potentially in contact if at least one’s BT device discovers that of the other.

Starting with raw GPS readings for each participant, we first discarded GPS readings with an accuracy radius larger than 10 m (32.8 ft) as being too inaccurate to allocate even approximate colocation estimates. For each participant, we used the median of their GPS readings within a duty cycle as the estimated geolocation of that participant. We then mapped the estimated GPS coordinates of latitude and longitude onto the Universal Transverse Mercator coordinates as the northing and easting with units of meters. For the sake of estimating interparticipant proximity, we used the Euclidean distance between the estimated geolocation for all pairs of participants within the same duty cycle as the estimated distance between pairs of participants. For each duty cycle, participants who lacked GPS readings within that duty cycle were considered as not being in contact with any of the other participants for the duration of that cycle.

#### Agent-Based SEIR Model

An agent-based SEIR simulation model was used to characterize pathogen transmission and describe the natural history of infection. The model assumed the following:

- There is no reinfection during the simulation period.
- The population is closed, and no births, deaths, and migrations occur during the simulation time horizon.
- The latent periods for diseases under consideration are similar to the incubation periods.
- During the infectious period, an infectious patient will have a constant hazard rate of transmission to every one of their currently contacted persons, normalizing passive shedding from active spread (eg, sneezing) over a contact period.
- There are no behavior changes in participants during the simulation period, conditional on the contact patterns measured. For small outbreaks, this is reasonable, but the COVID-19 pandemic has demonstrated the importance and magnitude of changes that can occur in hygienic personal protective behavior (eg, mask use) over the course of a pandemic.

We made use of a 4-fold duplication and concatenation of both GPS- and BT-inferred proximity contact data, such as successively replaying a movie, to allow the outbreak to run its course without running out of contact data.

All participants who connected to at least 1 other participant after filtering were included in the simulation (Table 2). Each simulation starts with 1 initially exposed participant. We conducted multiple simulations with different random seeds to account for stochastics. Each simulation began with a single participant with an infection of the corresponding disease. All active participants were the initially exposed participants in turn, for 50 realizations each.

During the initialization of each simulation realization, incubation period and infectious period were drawn uniformly from the minimum-maximum range of corresponding parameters, as presented in Table 3.

All diseases listed in Table 3 are investigated from a historical perspective, where estimates of the corresponding parameters are available.

**Table 2:** Number of participants with at least 1 contact within the 3 data sets (Saskatchewan Human Ethology Dataset [SHED]7, SHED8, AND SHED9). Proximity contacts are inferred from sensing data and vary by sensing modality and distance threshold.

	SHED7	SHED8	SHED9
BT8^a^, n	58	71	76
BT20, n	58	71	76
GPS8, n	49	63	66
GPS20, n	58	71	74

^a^The sensing modality of Bluetooth and GPS are combined with distance thresholds of 8 m (26.2 ft) and 20 m (65.6 ft) in rows. For example, BT8 stands for proximity contacts inferred from Bluetooth-sensed distance within the threshold of 8 m (26.2 ft).

**Table 3:** Disease parameter.

	R_0_	Incubation period, minimum to maximum	Infectious period minimum to maximum
COVID-19 (nonvariant)	2.2^a^ [57,58]	5.6-7.7 [57]	3-7 [57]
Influenza	3^b^ [59]	1-4 [60]	3-5 [60]
Norovirus	1.75 [61]	0.5-2 [62]	2-3 [62]
Measles	15^b^ [63]	10-12 [64]	8-11 [64]

^a^Derived as midpoint of reported range.

^b^Derived range from different reports.

#### Simulation Configuration

For each SHED study, after preprocessing, we obtained GPS- and BT-inferred proximity contact data with distance-equivalent RSSI thresholds of −80 dBm (corresponding to approximately 8 m or 26.2 ft) and −90 dBm (approximately 20 m or 65.6 ft). A group of simulations for each of the 4 diseases—namely, influenza, COVID-19, measles, and norovirus—was run. Within each group of simulations sharing the same derived proximity contact data and disease parameters, we iterated each of the active participants as the initially exposed patient with 50 realizations, where each realization has a different predetermined random seed, resulting in 170,400 realizations across all data sets and conditions.

In our agent-based SEIR model, at any given time during the simulation, each agent resides in one of the 4 disease states (susceptible, exposed, infectious, or removed). At the start of the simulation, all agents, except the initially exposed agent, are susceptible. The transition from susceptible to exposed has a probability *p* when exposed to proximity to an infectious agent. Such occurrences of exposure are characterized by a Poisson process with a mean interarrival time of 5 minutes. The value assumed for *p* is derived from the disease-specific *R_0_* and the average empirically observed frequency of population contacts. The timing, the duration, and the pair of agents involved in each proximity contact are given by the proximity contact data fed to the simulation. The transitions of exposed-to-infectious and infectious-to-removed are timeouts with timers set as the corresponding latent period and infectious period as initialized for each individual.

Simulations were run on 2 servers, each with an Intel Xeon CPU E5-2690 v2 and 503 GB memory. Models were created in AnyLogic (version 8.1.0; The AnyLogic Company) and exported to a stand-alone Java application with OpenJDK (version 1.8.0_252; The OpenJDK Community) as the runtime environment. Analysis was conducted in R software (version 4.0.2; R Foundation for Statistical Computing) with major packages, including *tidyverse* (version 1.3.0), *ggprah* (version 2.0.5), and *igraph* (version 1.2.6), and in Python (version 3.8.0; Python Software Foundation) with major packages, including *pandas* (version 1.2.0), *numpy* (version 1.20.2), and *scipy* (version 1.6.1).

#### Evaluate Impacts on Transmission Models

##### Overview

We used the attack ratio as the metric to evaluate the impact of proximate contact data on transmission models. The attack ratio is the proportion of the total population that gets infected throughout the simulation. Although the ABM-SEIR can produce many estimates for different disease parameters given proximate contact data, the attack rate and individual risk of infection was chosen for simplicity, accessibility, and to serve as single summary statistics [65,66].

##### Attack Rates

The core research question of this study was whether and to what extent the differences in GPS- and BT-based proximity detection would alter the contact network and therefore the implied attack rate. We considered the attack rate θ defined as in the following equation:



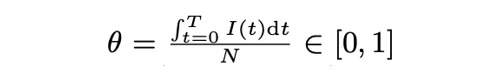



Where *I*(*t*) is the number of infectious persons at time *t*, *T* is the end time of the simulation instance, and *N* is the population size. The attack rate θ denotes the proportion of the population that is infected throughout the simulation instance. As the response variable (denoted as Θ) to the controlled variables of the disease or pathogen *M*, the initial infectious individual *ν* ∈ *V* = {*v_1_*,*v_2_*,...,*v_n_*}, *n* = ‖*V*‖ and collected proximity contact data D(*ω*,*ε*,*V*). For the proximity contact data D(*ω*,*ε*,*V*), *ω* ∈ {BT, GPS} is the sensor type, *ε* ∈ {8, 20} is the distance threshold of proximate contacts, and *V* is the underlying population. Therefore, with the ABM-SEIR model as P(∙) for a specific disease *M* and underlying population *V*, we can sample Θ∼P(Θ= θ |*ω*,*ε*,*M*,*V*) with simulation realizations. While the initial infectious individual *ν* has been known to impact the attack rate Θ, investigation of that impact lies outside the scope of this paper.

##### Welch *t* Test

Assuming disease *M* and an underlying population *V*, the choice of an initial infectious individual *ν* is independent of the data collection configuration (sensor type *ω* and proximate distance threshold *ε*). We were interested in the marginal probability, defined as in the following equation:



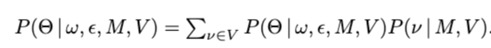



Limited by our knowledge of P(*ν*|*M*,*V*), we assumed the initial infectious individual *ν* is chosen with uniform probability from the underlying population *V*, that is, P(*ν*|*M*,*V*) =1 / ‖*V*‖. Consider θ as the sample mean from a sample, *X_i_* ∼ P(Θ|*ν* = *v_i_*,*ω*,*ε*,*M*,*V*), *i* = 1,..., ‖*V*‖, and we sampled by repeating ‖*V*‖ simulations iterating every individual of the population *V* as the initial infectious individual. According to the central limit theorem, samples of θ∼P(Θ|*ω*,*ε*,*M*,*V*) tend to be normally distributed to suffice the assumption of the Welch *t* test (2-tailed).

##### Pairwise *t* Test

Without assuming that the initial infectious individual *ν* is homogeneous among the underlying population *V*, that is, P(*ν*|*M*,*V*)=1/‖*V*‖, we could construct a pairwise *t* test by pairing the samples of attack rate having the same initial infectious individual *μ*, given sensor type *ω* and distance threshold *ε*, for each pair of disease *M* and underlying population *V*. In this case, we assumed the pairwise differences in the attack rate, such as for Θ*_i_*^BT8-GPS20^ = Θ^BT8^ – Θ^GPS20^, are normally distributed, where Θ^BT8^ ∼ P(Θ|*ν* = *v_i_*,*ω =* BT,*ε*= 8,*M*,*V*) and Θ^GPS20^ ∼ P(Θ|*ν* = *v_i_*,*ω* = GPS,*ε*= 20,*M*,*V*).

##### Kullback-Leibler Divergence of Individual Infection Risks

Given the sensor type, proximate distance threshold, disease, and underlying population, we estimated the individual infection risk based on the Laplacian-smoothed rate of being infected across realizations, denoted by *ρ_v∈V_*(*ω*,*ε*,*M*,*V*). The likelihood of being the most likely infected individual for an individual *v*∈ *V* follows P(*v*|*ω*,*ε*,*M*,*V*), which can be estimated by normalizing vector *ρ*={*ρ_v_*|*v*∈*V*}. The Kullback-Leibler (KL) divergence was used to summarize the differences between pairs of sensor type and proximate distance threshold (*ω*,*ε*) within blocks by disease and the underlying population. For disease *M* and the underlying population *V*, we have



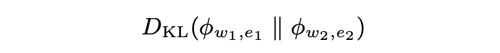



between sensing configurations (*w_1_*,*e_1_*) and (*w_2_*,*e_2_*), where



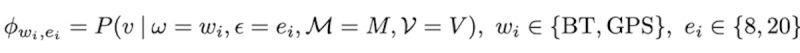



## Results

**Figure 1:**
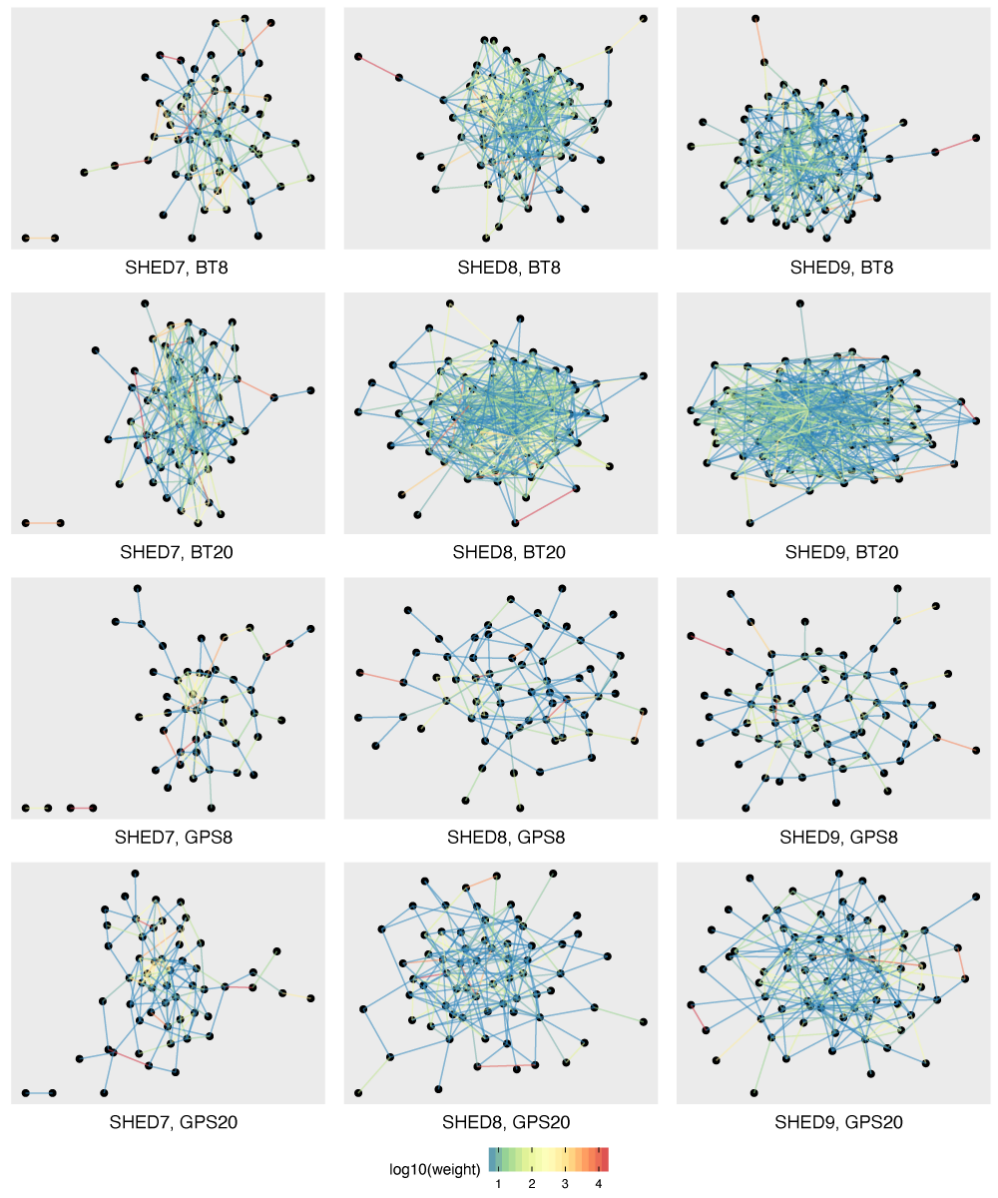
Stress layout of aggregated weighted contact network by underlying population and data source, with edges colored in log scale by weights. BT: Bluetooth; SHED: Saskatchewan Human Ethology Dataset.

While the agent-based simulation uses dynamic contacts, some insight can be gained by examining the aggregate contact network of participants in each study. Figure 1 shows the aggregate contact networks for SHED7, SHED8, and SHED9 using BT and GPS at 8- and 20-m thresholds. If a connection ever occurred between 2 nodes given the protocol, a corresponding edge is drawn in the network, with the color of the edge proportional to the total contact duration over the course of the experiment between those nodes. Reflecting the Pareto-like distribution of contact duration, colors move from blue (weakly connected) to red (strongly connected) on a logarithmic scale, consistent with other human network observations [67,68]. As expected, most nodes appear to have weak connections compared to the highly connected dyads and triads in the network. The BT networks are denser and more highly connected than their GPS counterparts, implying a greater potential for disease spread. There is a greater preponderance of weak edges in the BT data sets than in their corresponding GPS counterparts. There is a modest increase in the number of edges between the 8- and 20-m thresholds for each data set.

**Figure 2:**
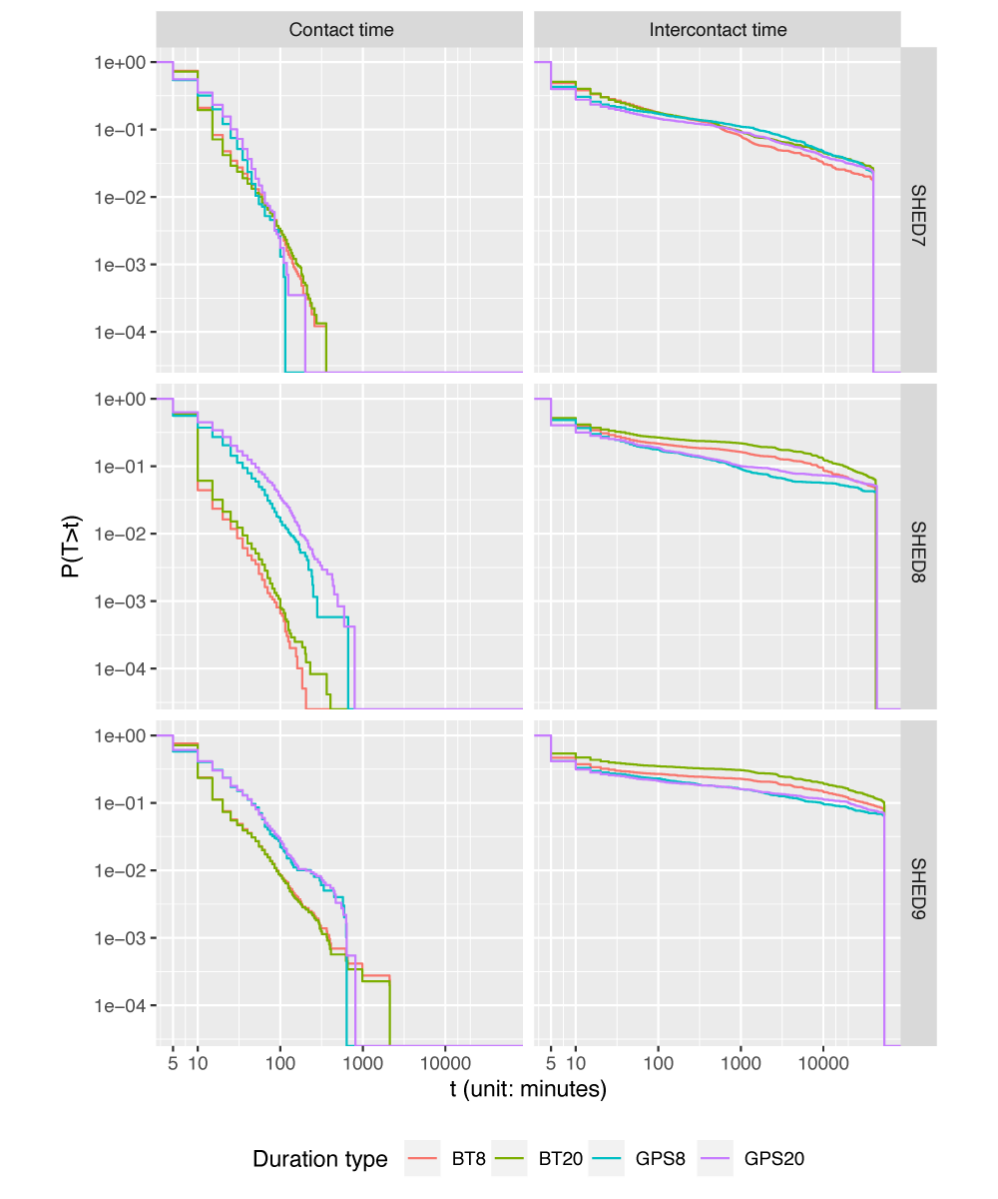
Empirical complementary cumulative distribution function of contact duration and intercontact time with different sources and distance thresholds. BT: Bluetooth; SHED: Saskatchewan Human Ethology Dataset.

Contact frequency (the rate at which contacts occur) and intercontact time (the time between contacts) are common aggregate distributions used to characterize contact data sets. Similar to many other data sets, both the BT and GPS demonstrate power law decay for the probability of contact duration and intercontact time (Figure 2). GPS-based contact detection tends to infer more and shorter-duration contacts but exhibits truncated tails. In SHED7 and SHED9, the tail truncation leads to fewer long-duration contacts (>600 min) than BT. The intercontact times are similar for all data sets, but the BT distributions are skewed more heavily toward longer intercontact times than in the case of GPS. In contrast, for SHED8 and SHED9, BT tracking detects notably fewer moderately long contacts (those in the range of 50 min to 600 min). This may be due to localization noise–induced false positives in the GPS data set skewing the apparent contact durations higher.

**Figure 3:**
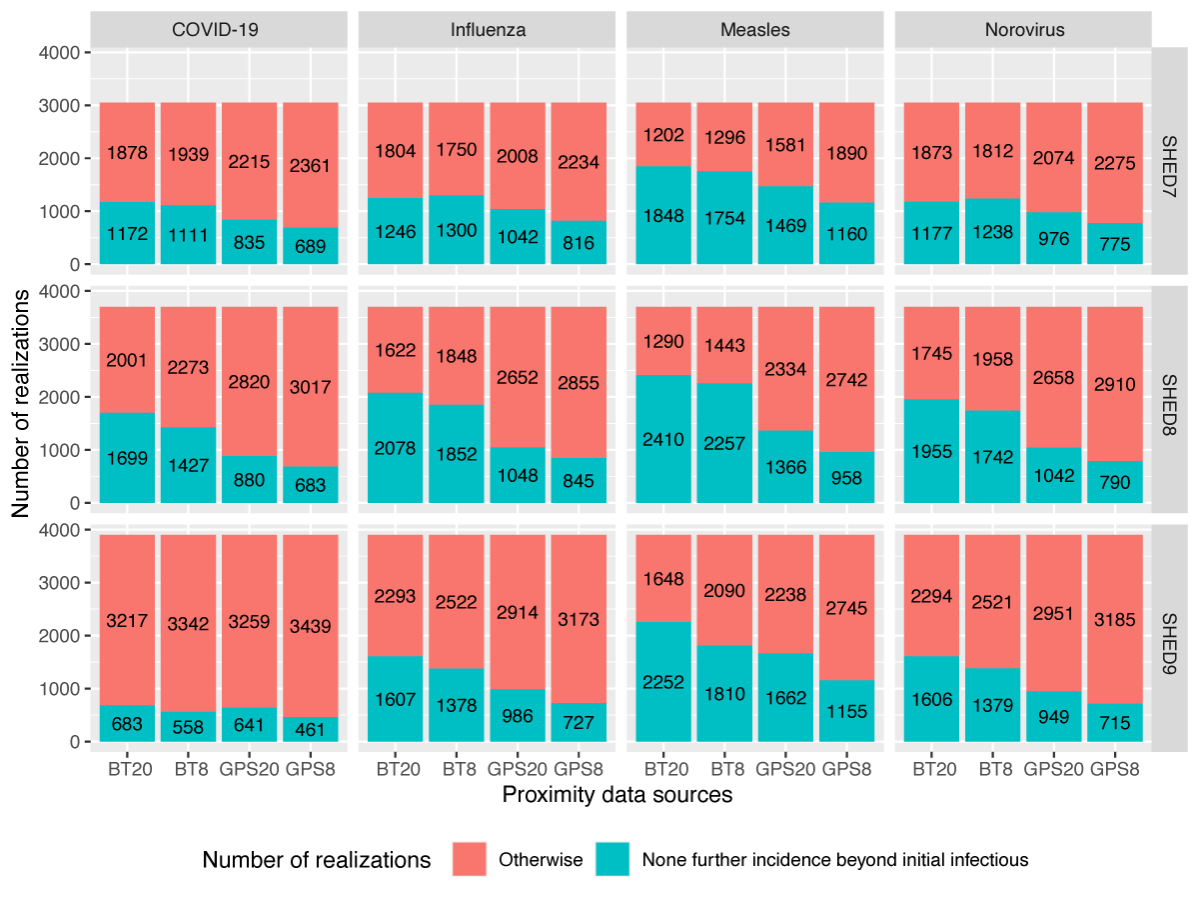
Number of realizations with or without further infections beyond initial infections. BT: Bluetooth; SHED: Saskatchewan Human Ethology Dataset.

After filtering the connections for the appropriate distance threshold (8 m and 20 m or approximately 26.2 ft and 65.6 ft), the agent-based simulation was run according to the protocol described in simulation configuration. Many runs do not produce an outbreak, with the initially exogenously infected individual being the only member of the network infected. This results in a zero-heavy bimodal distribution of cumulative infection counts per realization, with a Poisson spike at 0 cumulative endogenous infections (1 exogenous infection) and a second distribution describing the probability of an outbreak of a given size conditional on outbreak occurrence (ie, the probability of at least one endogenous infection). A stacked bar plot showing the ratio of runs in which further incidences beyond the initial infectious individual did or did not occur is shown in Figure 3. The figure clearly shows a higher likelihood of an outbreak occurring with the BT data, as expected from the aggregate network diagrams and aggregate contact duration and frequency plots. The consistent difference in the probability of outbreak occurrence between the 2 conditions is our first substantial indication that the two means of measuring contact are not equivalent. To determine the impact of each dynamic contact pattern on the outbreaks themselves, the trials in which no endogenous infection occurred were removed, and statistical analysis was conducted on the distribution of outbreak severity conditional on outbreak occurrence.

The Bonferroni-corrected Shapiro-Wilk test was passed for each of the 50 samples of attack rate, denoted by θ, for every pair of disease *M* and the underlying population *V*, except for COVID-19, with contact records collected via GPS using a distance threshold of 20 over SHED8. The results of the Bonferroni-corrected Welch *t* test are presented in Table 4.

**Table 4:** Bonferroni-corrected Welch *t* test for incidence number.

		*P* value					
		BT8-BT20^a^	BT8-GPS8	BT8-GPS20	BT20-GPS8	BT20-GPS20	GPS8-GPS20
**Norovirus**							
	SHED7	<.001	.05	<.001	<.001	<.001	<.001
	SHED8	<.001	<.001	<.001	<.001	<.001	<.001
	SHED9	<.001	<.001	<.001	<.001	.001	<.001
**Influenza**							
	SHED7	<.001	.12	<.001	<.001	<.001	<.001
	SHED8	<.001	<.001	<.001	<.001	<.001	.001
	SHED9	<.001	<.001	<.001	<.001	<.001	<.001
**COVID-19**							
	SHED7	<.001	.85	<.001	<.001	<.001	<.001
	SHED8	.04	<.001	<.001	<.001	<.001	.04
	SHED9	.001	<.001	>.99	<.001	<.001	<.001
**Measles**							
	SHED7	<.001	.003	<.001	<.001	>.99	<.001
	SHED8	<.001	<.001	<.001	<.001	<.001	<.001
	SHED9	<.001	<.001	<.001	<.001	<.001	<.001

^a^BT: Bluetooth.

Our predetermined α level was .05.

The results of Bonferroni-corrected pairwise *t* tests [69] between observed attack rates (having filtered out scenarios with 0 endogenous infections) across all simulation runs for a condition are presented in Table 5.

**Table 5:** Bonferroni-corrected pairwise *t* test for attack rate.

		*P* value					
		BT8-BT20^a^	BT8-GPS8	BT8-GPS20	BT20-GPS8	BT20-GPS20	GPS8-GPS20
**Norovirus**							
	SHED7	<.001	.25	<.001	<.001	<.001	<.001
	SHED8	.44	<.001	<.001	<.001	<.001	>.99
	SHED9	<.001	.01	.005	<.001	.008	<.001
**Influenza**							
	SHED7	<.001	>.99	<.001	<.001	<.001	<.001
	SHED8	<.001	<.001	<.001	<.001	<.001	>.99
	SHED9	<.001	.004	.02	<.001	<.001	<.001
**COVID-19**							
	SHED7	<.001	.73	<.001	<.001	<.001	<.001
	SHED8	.07	<.001	<.001	<.001	<.001	>.99
	SHED9	<.001	<.001	>.99	<.001	<.001	<.001
**Measles**							
	SHED7	<.001	>.99	<.001	<.001	.49	<.001
	SHED8	<.001	<.001	<.001	<.001	<.001	.002
	SHED9	<.001	<.001	<.001	<.001	<.001	<.001

^a^BT: Bluetooth.

These results confirm our hypothesis that BT- and GPS-based contact histories induce significantly different estimates of total disease burden across multiple simulated realizations. The primary comparisons are the BT8-GPS8 and the BT20-GPS20, with the others included for completeness. For SHED7 BT8-GPS8, the results are not significant. For all other diseases and data sets, the results are statistically significantly different. In the case of BT20-GPS20, all results are significantly different, with the exception of the SHED7 measles. While we suspected that the infectiousness of the disease would impact simulated outcomes, the results seem to be dominated by differences in the data set and contact measurement modality. Looking at the impact of resolution, some combinations of data set and disease are not significantly different, but for the most part, increasing the threshold increases the number of contacts, driving differences in simulated outcomes. The exception to this general rule seems to be SHED8 GPS8-GPS20, where increasing the threshold did not significantly alter the outcomes for most diseases and only marginally for measles.

**Figure 4:**
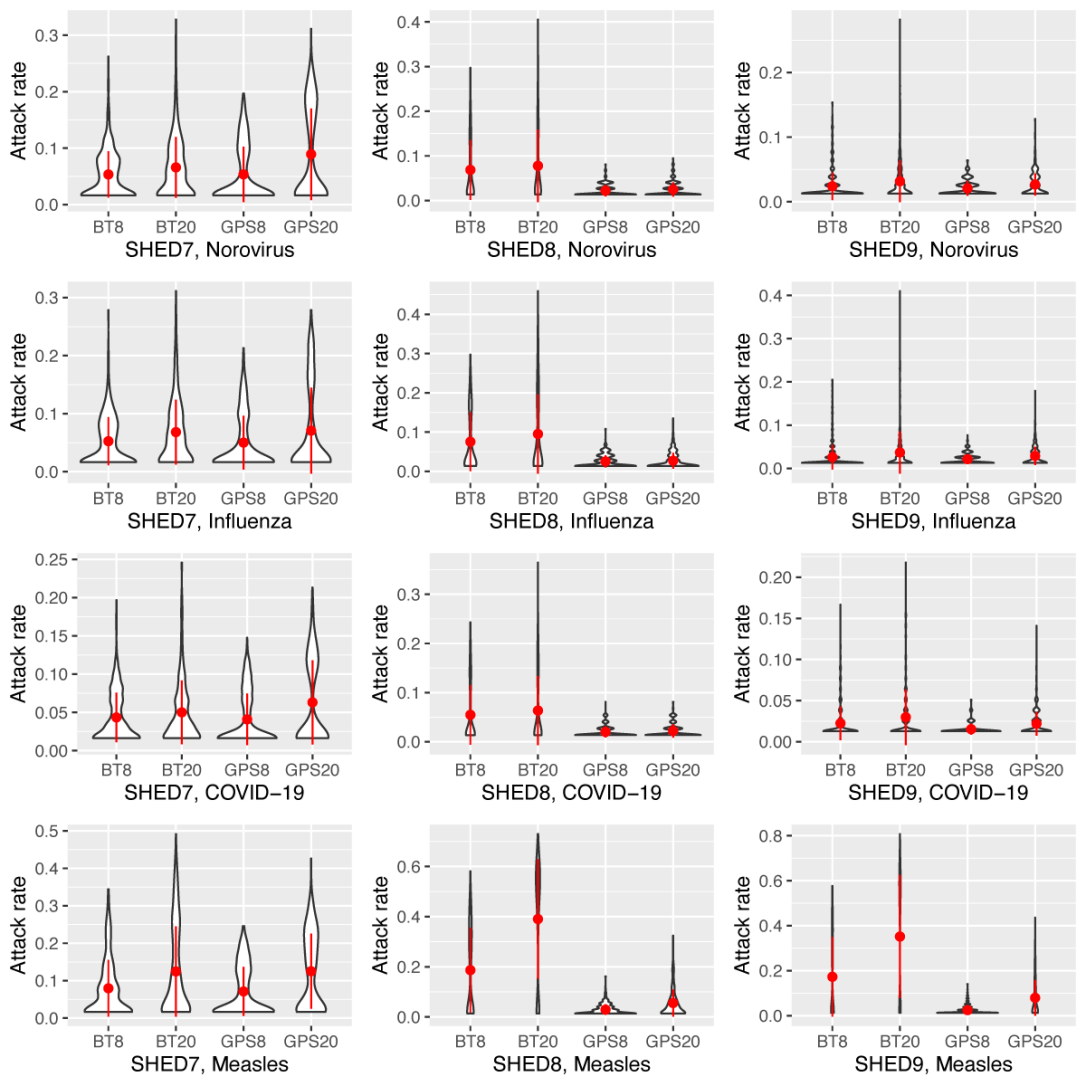
Distribution of the attack rate (filtered out 0) for data collections and diseases. BT: Bluetooth; SHED: Saskatchewan Human Ethology Dataset.

Figure 4 shows the violin plots for the attack rates over each realization across all simulated conditions and provides insight into the statistical results from Table 5. SHED7 consistently has lower attack rates for all diseases, with a smaller variance and mean than other data sources. The limited attack rate likely drives the similarity between the BT and the GPS. The denser SHED8 and SHED9 networks have substantially larger variance, leading to significant differences between the measurement modality conditions. The highly contagious measles virus, in particular, exhibits marked differences within the SHED8 and SHED9 data sets. In general, BT contact patterns have longer tails, indicating a greater possibility of larger outbreaks throughout the population. In cases where a substantial probability mass is contained in the tail, the median is also drawn higher, as in SHED8 with BT20 for measles.

**Figure 5:**
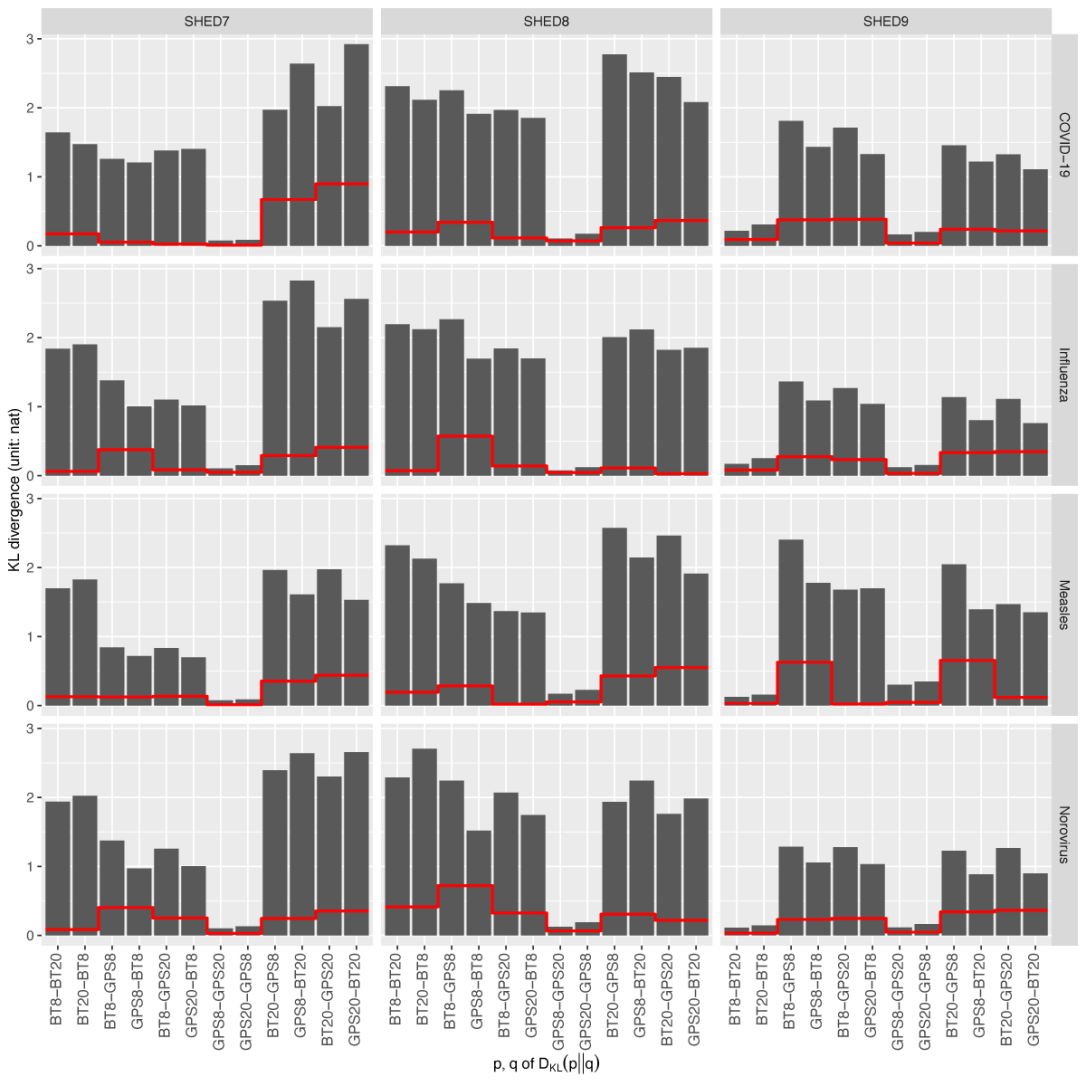
Kullback-Leibler divergence of individual infection risks.

Figure 5 shows the KL divergence on individual infection risks within blocks of disease and the underlying population. The individual infection risks are reflected by the likelihood of being the most likely infected individual between different sensing configurations, where a sensing configuration is a pair of selected sensor types and proximate distance thresholds. The distance threshold of proximate contact does not appear to impact GPS-collocated inferred proximity contacts in terms of individual infection risks, regardless of the underlying population. This invariance to distance thresholds suggests that the primary bottleneck lies in the GPS-colocation method’s inability to identify exact proximity contacts among a group of collocated individuals. Meanwhile, the BT-beaconing method may capture proximity contacts at certain distance thresholds (such as for SHED7 and SHED8), which can be important when considering droplet-based pathogen transmission. There seems to be a lower magnitude of KL divergence for BT8-BT20 and BT20-BT8. The KL divergence among pairs of different sensor types is similar regardless of the distance thresholds of proximate contact, suggesting that BT beaconing and GPS colocating collect different proximity contacts regardless of the resolution of the distance thresholds of proximate contacts. The magnitude of asymmetric |*D*_KL_(*p*‖*q*) – *D*_KL_(*q*‖*p*)| shown in red lines is lower than either *D*_KL_(*p*‖*q*) or *D*_KL_(*q*‖*p*), indicating that the asymmetry of the KL divergence is not impairing our aforementioned analyses.

## Discussion

### Disparate Results From GPS- and BT-Based Contact Tracking

Our results clearly indicate that GPS- and BT-based contact tracking yield disparate results for the same cohort under measurement. The ground truth contact network, while unknown, was the same for each data set—it was the same set of participants carrying a single phone measuring both quantities. Both BT- and GPS-derived contact measurements are estimates of the underlying contact pattern, admitting false positives (eg, BT contacts through a wall) and negatives (eg, a missed GPS contact because it occurred in an area of poor satellite reception). GPS-based contact tracking identifies fewer shorter contacts, leading to a significant decrease in expected outbreak intensity and the number of outbreaks, potentially because both participants need to have a sufficiently good location fix to estimate colocation. The denser contacts reported by BT-based contact tracking led to a higher probability of an outbreak and larger outbreaks, resulting in significantly different attack rates for most data sets and diseases. While there were conditions under which no significant differences were observed across the data collection modalities (particularly for SHED7 BT8-GPS8), differences were often significant enough to encourage caution in the uptake and interpretation of these sensed contact networks. GPS8 tends to underestimate the attack rate relative to the others (BT8, BT20, and GPS20), indicating the general inability of GPS colocating to capture proximate contacts within a short distance. Sensing configurations tend to estimate similar attack rates for infectious diseases without a comparatively high *R_0_* in a more distant underlying population, except for SHED9-measles. Our study cannot conclusively determine if the higher outbreak frequency and size in BT-derived networks is due to false positives in BT or false negatives in GPS, but based on the precision of commodity GPS receivers and their propensity to lose signal in large buildings, we suspected that the observed disparities are predominantly driven by GPS false negatives. If this suspicion is warranted, GPS location–based proximity measurement should be used in epidemiological simulations with caution and in a fashion that anticipates and accounts for the fact that the data collection modality used may be systemically underestimating contact. This is particularly true for the short contacts outside of normal contact networks that drive mixing.

The significance results were relatively insensitive to differences in simulated disease impacting differences in GPS and BT, but the data collection modality induced fewer differences in the results for less contagious diseases, such as seasonal influenza, than for more contagious diseases, such as measles. It is possible that weakly contagious diseases might not demonstrate differences, as outbreaks would be rare and limited in both GPS and BT networks. These findings hold for both a nominal 8-m and 20-m threshold for determining if contact has occurred. The thresholds chosen are already judicious and indicate participants being close enough during a measured portion to have come into close contact during a sensor sleep period, rather than explicitly detecting close contact. Comparing the within-sensor outcomes, the contact threshold impacted the simulated attack rate for most cases, with the exception of SHED8.

We used a stylized, agent-based SEIR model to determine the attack rate using both BT- and GPS-inferred temporal contact patterns. The stylized nature of the simulation implies that the results should be generally correct, but that more detailed models may diverge in the magnitude of the differences observed. SHED7, SHED8, and SHED9 are interesting data sets due to the multiple sensor modalities, but they are also highly biased, being drawn from a university social science participant pool comprised primarily of undergraduate students in the social and physical sciences. GPS or BT data from other demographics will almost certainly have different contact patterns, leading to different outcomes. At one extreme, institutionalized individuals (eg, in incarceration facilities or care homes) have limited mobility and would be expected to have much more convergent GPS and BT contact patterns. Perhaps not surprisingly, some of the worst COVID-19 outbreaks occurred in these institutional settings. Similarly, we analyzed 4 relatively contagious diseases and ignored diseases where a specific type of contact initiates infection, such as sexually transmitted or blood-borne diseases, or where disease propagation is slow or exhibits prolonged latent periods, such as with tuberculosis. As the definition of contact for such excluded diseases is substantially different from those analyzed in this study, the difference between GPS and BT contact patterns may be more or less pronounced. The process we have used to evaluate the differences should generalize to any contagious disease or measured contact pattern and can be used to evaluate the impact of novel contact detection algorithms or other novel diseases such as COVID-19 variants of concern.

While this study has made several meaningful contributions to the literature, particularly in highlighting divergent attack rates for GPS and BT measurements of the same underlying contact network, it is subject to notable limitations. We used 3 data sets drawn from a social sciences participant pool at our institution. These data sets included individuals who were often unknown to each other and likely produced more diffuse data sets than would have been expected had we used snowball or respondent-driven sampling or other socially connected recruiting techniques. Running a similar analysis on other data sets could provide more broadly generalizable or representative results. However, for reasonable privacy reasons, public data sets containing both GPS and BT records are not available, requiring additional measurement effort to extend this analysis. We used an agent-based SEIR model because it provided the most direct link between the data and the simulated diseases. We chose the stylized SEIR model to emphasize the role of evolving contact networks in other disease dynamics. These results could be extended to include more sophisticated disease models and compared against compartmental transmission models grounded in aggregate representations of the underlying contact network. The COVID-19 pandemic has driven innovation in contact tracing, and new measurement techniques based on dongles, beacons, or badges are now readily available. A similar analysis including these data sources could be valuable. Finally, we constrained our analysis to 4 canonical contagious diseases from a historical perspective with relatively well-parameterized behaviors. However, novel diseases will have novel disease parameters. An exploratory simulation study that outlined how diseases might be expected to behave over these contact networks using, for example, a random-walk through parameter space might be valuable in predicting new variants, existing diseases, or new diseases emerging from animal reservoirs.

### Conclusions

Epidemiological models of disease propagation are an important tool in controlling and containing epidemic outbreaks. These models rely on the accurate measurement of key biological and behavioral parameters to ground the simulation results. Quantifying the characteristics of dynamic contact networks is a particularly challenging aspect of grounding these simulations. The significant differences in the predicted outcomes for contact networks demonstrated here between GPS- and BT-based contact tracking highlight the difficulty of grounding these simulations. Because of the nature of our data, we know that the contact networks being sought via measurement by BT and GPS should have been identical, as they corresponded to the same device held by the same individual as they went about their lives. The fact that the resulting contact networks and predicted attack rates were different indicates that these modalities are not interchangeable and that caution should be exercised by modelers employing these measures. While BT and GPS data provide more precise measurements than traditional surveys, they are still prone to error and disparate estimates of the underlying network structure and dynamics.

## Abbreviations

ABM: agent-based model
BT: Bluetooth
dBm: decibel-milliwatts
KL: Kullback-Leibler
RSSI: Received Signal Strength Indicator
SEIR: susceptible-exposed-infectious-removed
SHED: Saskatchewan Human Ethology Dataset

## Data Availability

Data cannot be made publicly because participant informed consent does not allow disclosure and because anonymized longitudinal location data can be reidentified through crosslinking by those skilled in spatial analysis. Data can be made available through a joint application to both the requester’s and the University of Saskatchewan ethics boards, outlining the intended secondary use of the data and processes for ensuring that reidentification does not occur. The latest contact information for the research ethics board (REB) can be found on the website of the University of Saskatchewan. Currently, the web page for the REB is available [70].
